# Echocardiographic epicardial fat thickness is a predictor for target vessel revascularization in patients with ST-elevation myocardial infarction

**DOI:** 10.1186/s12944-016-0371-8

**Published:** 2016-11-16

**Authors:** Jin-Sun Park, You-Hong Lee, Kyoung-Woo Seo, Byoung-Joo Choi, So-Yeon Choi, Myeong-Ho Yoon, Gyo-Seung Hwang, Seung-Jea Tahk, Joon-Han Shin

**Affiliations:** Department of Cardiology, Ajou University School of Medicine, 164 Worldcup-ro, Yeongtong-gu, Suwon, 16499 Korea

**Keywords:** Epicardial adipose tissue, Myocardial Infarction, Prognosis

## Abstract

**Background:**

The amount of epicardial adipose tissue (EAT) has been demonstrated to correlate with the severity of coronary artery disease (CAD) and the CAD activity.

The aim of this study is to assess the impact of EAT on long term clinical outcomes in patients with ST elevation myocardial infarction (STEMI) after percutaneous coronary intervention (PCI).

**Methods:**

We analyzed the data and clinical outcomes of 761 patients (614 males, 57 ± 12 year-old) with STEMI who underwent successful primary PCI from 2003 to 2009. All patients were divided into two groups: thick EAT group, EAT ≥ 3.5 mm and thin EAT group, EAT < 3.5 mm. The primary end points were all-cause death, recurrent MI, target vessel revascularization (TVR) and major cardiac adverse events (MACEs), composite of all-cause death, recurrent MI and TVR, within 5 years.

**Results:**

Median and mean EAT of 761 patients were 3.3 mm and 3.6 ± 1.7 mm, respectively. Mean follow up period was 46 ± 18 months. MACE-free survival rate in the thick EAT group was significantly lower than in the thin EAT group (log-rank *P* = 0.001). The event-free survival rate of all-cause death of the thick EAT group was significantly lower than that of the thin EAT group (log-rank *P* = 0.005). The TVR-free survival rate in the thick EAT group was significantly lower than in the thin EAT group (log-rank *P* = 0.007). The event-free survival rate of recurrent MI were not significantly different between the groups (log-rank *P* = 0.206). In the Cox’s proportional hazard model, the adjusted hazard ratio of thick EAT thickness for TVR was 1.868 (95% confidence interval 1.181–2.953, *P* = 0.008).

**Conclusion:**

This study demonstrates that the EAT thickness is related with long term clinical outcome in patients with STEMI. The EAT thickness might provide additional information for future clinical outcome, especially TVR.

## Background

The amount of epicardial adipose tissue (EAT) has been demonstrated to correlate with the severity of coronary artery disease (CAD) and the CAD activity [1, 2]. Our previous study demonstrated that EAT thickness was closely related with short term clinical outcomes in patients with significant CAD after successful percutaneous coronary intervention (PCI) [3]. There has been few study demonstrating the effect of EAT on long term clinical outcomes in patients with ST elevation myocardial infarction (STEMI).

Systemic inflammation has a pivotal role in adverse outcomes in patients with STEMI [4–6]. As the metabolically active EAT could amplify inflammation [7, 8], the additional local inflammation of EAT might influence the adverse outcomes. Thus, EAT quantification might provide additional information for future events in patients with STEMI.

The aim of this study is to assess the impact of EAT on long term clinical outcomes in patients with STEMI after successful PCI.

## Methods

The study population consisted of patients admitted to Ajou University Medical Center for STEMI from 2003 to 2009. We consecutively enrolled 30-day survivors after STEMI, who underwent successful PCI. Successful PCI was defined as thrombolysis in myocardial infarction trial (TIMI) grade 3 flow and < 30% residual stenosis in the infarct related artery after primary percutaneous coronary intervention (PCI). The medical records of all patients were retrospectively reviewed. This study was approved by the Ajou University Hospital Institutional Review Board (approval number: AJIRB-MED-MDB-12-277). We excluded patients from the study if they had history of prior revascularization. We also excluded patients if the LV dysfunction was caused by any of the following: predisposing cardiomyopathy, severe valvular heart disease including symptomatic aortic stenosis, more than moderate aortic and mitral regurgitation. As active inflammation, such as infection or systemic autoimmune disease, often related to increased EAT [9, 10], we also excluded patients from the study if they had active inflammation.

Two-dimensional transthoracic echocardiography was performed within 48 h of primary PCI. Recordings of three cycles of the two-dimensional parasternal long-axis view were obtained. Images were enlarged for better visualization and accurate measurement of EAT thickness. EAT thickness was measured on the free wall of the right ventricle (RV) in a still image at end diastole on the parasternal long-axis view. EAT thickness was measured at the point on the free wall of the RV at which the ultrasound beam was oriented perpendicularly to the aortic annulus [11–14]. The anterior echo-lucent space between the linear echo-dense parietal pericardium and the RV epicardium was considered to be EAT. We measured the thickest point of EAT in each cycle. The average value of the EAT thickness was calculated. All patients were divided into two groups: thick EAT group, EAT thickness ≥ 3.5 mm and thin EAT group, EAT thickness < 3.5 mm. As the median value of EAT thickness in patients with unstable presentation or significance of coronary artery disease was 3.5 mm in our previous studies [1, 2, 15], we chose 3.5 mm as a reference. The primary end points were all-cause death, recurrent MI, target vessel revascularization (TVR) and major cardiac adverse events (MACEs), composite of all-cause death, recurrent MI and TVR, within 5 years. Recurrent MI was defined according to the universal definition of MI [16]. The TVR was defined as clinically indicated percutaneous or surgical revascularization of the index vessel during follow-up. At 5 years after index STEMI, follow-up data were obtained by reviewing medical records and/or telephone interview with patients.

SPSS 13.0 statistical software package (SPSS, Chicago, Illinois, USA) was used for all calculations. Data are shown as the mean ± standard deviation for continuous variables and as numbers and percentages for categorical variables. Comparisons were conducted by unpaired Student’s *t* test for continuous variables and Pearson chi-square test for categorical variables. Event free survival analysis for patients in these groups was performed using the Kaplan-Meier method, and the differences between groups were assessed by the log-rank test. To assess the adjusted relative hazard ratio (HR) of EAT thickness to the study end points, Cox’s proportional hazard model was used with potential variables associated with clinical outcomes. Adjusted covariates for the Cox’s proportional hazard model were age, gender, diabetes mellitus, hypertension, smoking, dyslipidemia, Killip classification, left ventricular ejection fraction and thick EAT thickness (≥3.5 mm). The results of Cox’s regression analysis were expressed as adjusted HRs and their 95% confidence intervals (CI) for clinical outcomes. Multivariate logistic regression analysis was performed to assess the effect of EAT thickness on clinical outcomes. Null hypotheses of no difference were rejected if *P* values were < 0.05.

## Results

Total 761 patients (614 males, 57 ± 12 year-old) were enrolled. Median and mean EAT of 761 patients were 3.3 mm and 3.6 ± 1.7 mm, respectively. Three hundred fifty patients (46%) were included in the thick EAT group and the others were included in the thin EAT group (411 patients, 54%). Patients in the thick EAT group were older (61 ± 12 vs. 54 ± 12 year-old, *P* < 0.001), had less males (72 vs. 88%, *P* < 0.001), less smokers (59 vs. 69%, *P* = 0.003) and more history of hypertension (45 vs. 34%, *P* = 0.002) than the patients in the thin EAT group. The laboratory findings did not show any significant difference at index STEMI. There was no significant difference in medical treatments between the groups (Table 1). Baseline data of angiography and echocardiography are listed in Table 2. The baseline characteristics of angiography were similar in both groups. There was no significant difference in the results of echocardiographic findings in both groups.

**Table 1:** Baseline clinical characteristics

Variables	EAT ≥ 3.5 mm	EAT < 3.5 mm	*P* value
	(*n* = 350)	(*n* = 411)	
Age (year-old)	61 ± 12	54 ± 12	<0.001
Men, n (%)	253 (72)	361 (88)	<0.001
BMI (kg/m^2^)	24 ± 3	25 ± 3	0.331
Medical History			
Hypertension, *n* (%)	157 (45)	140 (34)	0.002
Diabetes Mellitus, *n* (%)	81 (23)	77 (19)	0.138
Dyslipidemia, *n* (%)	25 (7)	32 (8)	0.737
Previous CVA, *n* (%)	11 (3)	12 (3)	0.858
Smoking, *n* (%)	206 (59)	285 (69)	0.003
LDL cholesterol (mg/dl )	106 ± 32	102 ± 34	0.079
hs-CRP (mg/L)	1.3 ± 3	1.1 ± 3	0.46
Killip class			
Killip class 3, *n* (%)	26 (7)	27 (7)	0.643
Killip class 4, *n* (%)	12 (3)	16 (4)	0.734
Medication at discharge			
Beta-blocker, *n* (%)	234 (67)	292 (71)	0.214
ACE inhibitor, *n* (%)	230 (66)	257 (63)	0.362
ARB, *n* (%)	110 (31)	135 (33)	0.677
CCB, *n* (%)	61 (17)	64 (16)	0.491
statin, *n* (%)	245 (70)	277 (67)	0.441

*EAT* epicardial adipose tissue, *BMI* body mass index, *CVA* cerebrovascular accident, *LDL* low-density lipoprotein, *hs-CRP* high sensitivity C-reactive protein, *ACE* angiotensin-converting enzyme, *ARB* angiotensin receptor blocker, *CCB* calcium channel blocker

**Table 2:** Baseline angiographic and echocardiographic characteristics

Variables	EAT ≥ 3.5 mm	EAT < 3.5 mm	*P* value
	(*n* = 350)	(*n* = 411)	
Culprit lesion			
LAD, *n* (%)	193 (55)	224 (55)	0.86
LCX, *n* (%)	32 (9)	30 (7)	0.355
RCA, *n* (%)	124 (35)	153 (37)	0.608
LM, *n* (%)	0 (0)	4 (1)	0.045
Coronary Artery Disease			
1 vessel disease, *n* (%)	145 (41)	191 (46)	0.163
2 vessel disease, *n* (%)	118 (34)	128 (31)	0.451
3 vessel disease, *n* (%)	87 (25)	92 (22)	0.423
PCI			
BMS, *n* (%)	68 (19)	77 (19)	0.566
DES, *n* (%)	276 (79)	331 (81)	0.808
Echocardiographic findings			
LVEDD (mm)	50 ± 5	50 ± 5	0.159
LVESD (mm)	34 ± 6	34 ± 6	0.3
LVEDV (mL)	87 ± 19	88 ± 23	0.789
LVESV (mL)	45 ± 14	45 ± 14	0.885
LVMI (g/m^2^)	115 ± 28	114 ± 32	0.554
LVEF (%)	52 ± 10	51 ± 10	0.861
WMSI	1.51 ± 0.34	1.52 ± 0.34	0.751
Ischemic MR, *n* (%)	8 (2)	7 (2)	0.565

*EAT* epicardial adipose tissue, *LAD* left anterior descending artery, *LCX* left circumflex artery, *RCA* right coronary artery, *LM* left main artery, *PCI* primary coronary intervention, *BMS* bare metal stent, *DES* drug eluting stent, *LVEDD* left ventricular end diastolic dimension, *LVESD* left ventricular end systolic dimension, *LVEDV* left ventricular end diastolic volume, *LVESV* left ventricular end systolic volume, *LVMI* left ventricular mass index, *LVEF* left ventricular ejection fraction, *WMSI* wall motion score index, *MR* mitral regurgitation

Patients were followed up for 46 ± 18 months after index STEMI. The MACEs occurred in 142 patients (19%). Of 761 patients, 62 patients (8%) died, 29 patients (4%) experienced recurrent MI and 81 patients (11%) needed TVR. In the thick EAT group, more MACEs occurred (23 vs. 15%, *P* = 0.004), more patients died (11 vs. 6%, *P* = 0.006) and more patients needed TVR (13 vs. 9%, *P* = 0.042). Rate of recurrent MI was not significantly different between the groups (7 vs. 4%, *P* = 0.189).

**Fig. 1:**
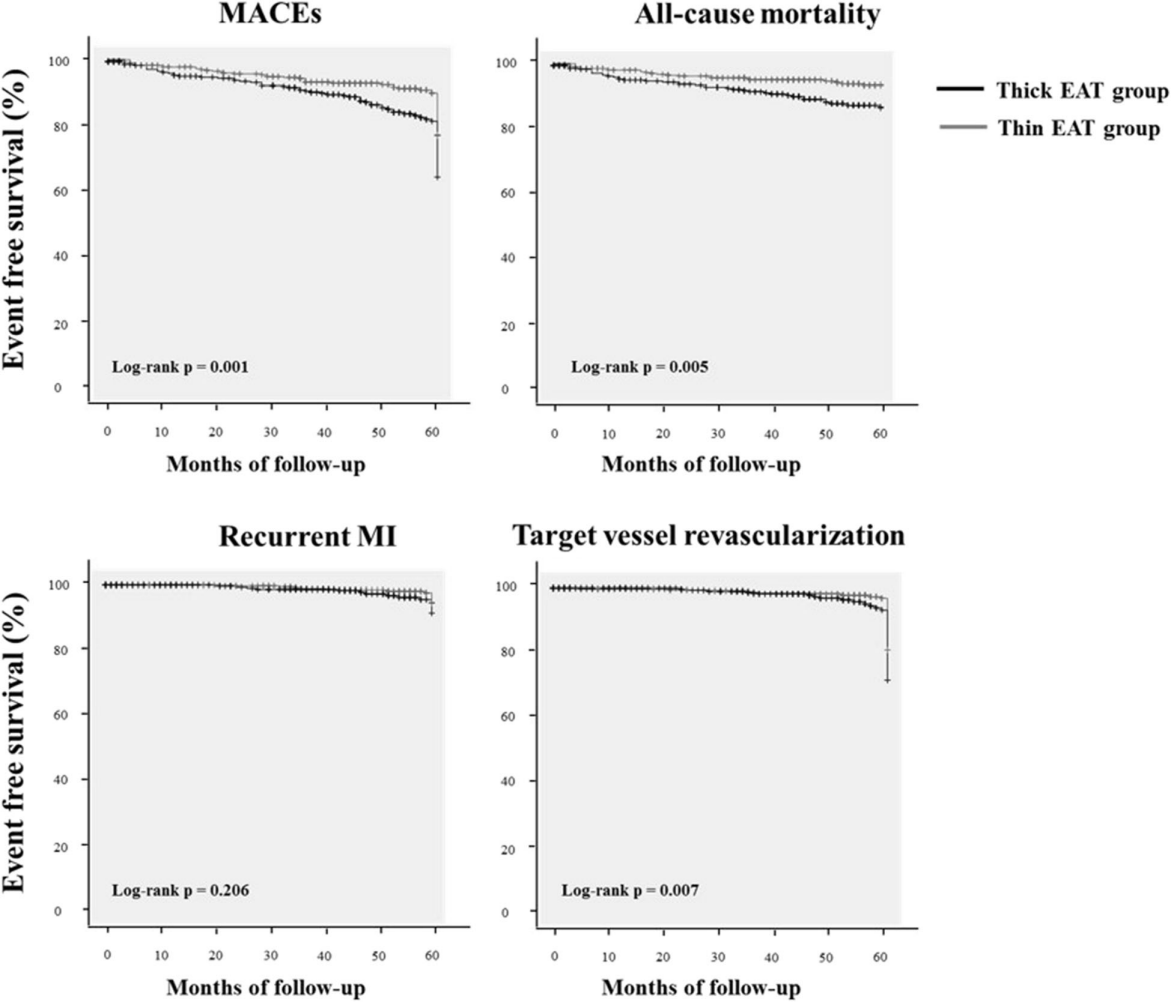
Kaplan-Meier survival curves for free of adverse outcomes in the thick epicardial adipose tissue (EAT) group and the thin EAT group. MACEs, major adverse cardiovascular events; EAT, epicardial adipose tissue; MI, myocardial infarction

Kaplan-Meier analysis (Fig. 1) revealed that MACE-free survival rate in the thick EAT group was significantly lower than in the thin EAT group (log-rank *P* = 0.001). The survival of the thick EAT group was significantly worse than the thin EAT group (log-rank *P* = 0.005). In addition, The TVR-free survival rate in the thick EAT group was significantly lower than in the thin EAT group (log-rank *P* = 0.007). The event-free survival curves for freedom of recurrent MI were not significantly different between the groups (log-rank *P* = 0.206).

The multivariate survival analysis using Cox’s regression model are reported in Table 3. In the Cox’s proportional hazard model, age was strongly related to MACEs (HR 1.036, 95% CI 1.020–1.054, *P* < 0.001). Age (HR 1.098, 95% CI 1.070–1.128, *P* < 0.001), Killip classification (HR 1.329, 95% CI 1.031–1.714, *P* = 0.028) and LVEF (HR 0.971, 95% CI 0.948–0.995, *P* = 0.02) were related to all cause of death. The adjusted HR of thick EAT thickness for TVR was 1.868 (95% CI 1.181–2.953, *P* = 0.008). In a multivariate regression model, the EAT thickness was independently associated with increased risk for all-cause mortality and TVR (HR 6.394, 95% CI 1.779–11.009, *P* = 0.007 and HR 5.846, 95% CI 1.576–10.117, *P* = 0.007, Table 4).

**Table 3:** Cox’s regression analysis for the adverse outcomes

Variables	Adjusted Hazard ratio (95% CI)	*P* value
MACEs		
Age	1.036 (1.020–1.054)	<0.001
Gender	0.997 (0.626–1.587)	0.99
Hypertension	0.945 (0.668–1.336)	0.747
Diabetes	1.318 (0.895–1.942)	0.162
Dyslipidemia	0.361 (0.114–1.147)	0.084
Smoking	1.289 (0.862–1.928)	0.217
Killip classification	1.135 (0.937–1.375)	0.197
LVEF	0.993 (0.977–1.009)	0.379
EAT thickness ≥ 3.5 mm	1.382 (0.973–1.961)	0.07
All-cause mortality		
Age	1.098 (1.070–1.128)	<0.001
Gender	0.935 (0.484–1.805)	0.842
Hypertension	1.022 (0.611–1.71)	0.934
Diabetes	1.501 (0.832–2.705)	0.177
Dyslipidemia	0 (0–3.801)	0.97
Smoking	1.768 (0.952–3.282)	0.71
Killip classification	1.329 (1.031–1.714)	0.028
LVEF	0.971 (0.948–0.995)	0.02
EAT thickness ≥ 3.5 mm	1.110 (0.637–1.934)	0.712
Recurrent MI		
Age	1.001 (0.965–1.038)	0.966
Gender	0.64 (0.207–1.761)	0.356
Hypertension	0.624 (0.27–1.445)	0.271
Diabetes	0.952 (0.359–2.527)	0.921
Dyslipidemia	0.657 (0.087–4.985)	0.685
Smoking	1.767 (0.661–4.72)	0.256
Killip classification	0.838 (0.472–1.487)	0.546
LVEF	1.017 (0.982–1.053)	0.351
EAT thickness ≥ 3.5 mm	1.661 (0.771–3.577)	0.195
TVR		
Age	0.999 (0.978–1.021)	0.932
Gender	1.389 (0.674–2.86)	0.932
Hypertension	0.794 (0.493–1.28)	0.794
Diabetes	1.624 (0.987–2.672)	0.056
Dyslipidemia	0.989 (0.35–2.796)	0.984
Smoking	1.219 (0.7–2.123)	0.484
Killip classification	1.050 (0.792–1.391)	0.734
LVEF	1.007 (0.986–1.027)	0.532
EAT thickness ≥ 3.5 mm	1.868 (1.181–2.953)	0.008

*MACEs* major adverse cardiovascular events, *LVEF* left ventricular ejection fraction, *EAT* epicardial adipose tissue, *MI* myocardial infarction, *TVR* target vessel revascularization, *CI* confidence interval

**Table 4:** Multivariate logistic regression analysis of the epicardial adipose tissue thickness for adverse outcomes

Variables	Hazard ratio (95% CI)	*P* value
MACEs	0.03 (-0.039–0.1)	0.389
All-cause mortality	6.394 (1.779–11.009)	0.007
Recurrent MI	1.031 (-5.659–7.721)	0.762
TVR	5.846 (1.576–10.117)	0.007

*MACEs* major adverse cardiovascular events, *MI* myocardial infarction, *TVR* target vessel revascularization, *CI* confidence interval

## Discussion

The present study demonstrated the close relationship between EAT thickness by echocardiography and increased rate of adverse clinical outcomes in patients with STEMI who underwent successful PCI.

Accompanied by the concept of early intervention in patients with STEMI and improvements in medications, cardiovascular mortality after STEMI has declined [17]. Although the number of patients with severe LV dysfunction, presence of residual myocardial ischemia or extent electrical instability, known as predictors for cardiovascular mortality [18], have significantly decreased, risk stratification after AMI still remains important [19]. Technical improvements in coronary intervention also resulted in absence of residual myocardial ischemia or extent electrical instability. In the present study, none had severe LV dysfunction, residual myocardial ischemia or extent electrical instability owing to early intervention. We could not find effect of angiographic and echocardiographic parameters on clinical outcomes, except LVEF on all cause of death, resulting from relatively good global and regional LV function and complete revascularization of the study population. Although these well-known predictors are still valid, additional predictors after STEMI should be evaluated for improving clinical outcomes.

The infarcted cardiomyocytes results in release of their intracellular contents and initiates an intense inflammatory reaction [20]. Necrotic cells and damaged extracellular matrix release endogenous damage-associated molecular pattern molecules (DAMPs). In the injured myocardium, DAMPs may potently stimulate inflammatory cascades by stimulating the toll-like receptor (TLR) family [21–23]. Generation of reactive oxygen species (ROS) in the infarcted cardiomyocytes also directly induces pro-inflammatory cascades, resulting in generation of active interleukin (IL)-1, the prototypical pro-inflammatory cytokines that drives expression of inflammation mediators [20, 24]. Most of studies found that systemic inflammation independently predicts MACE [4–6].

In STEMI, inflammatory cells are functionally activated [25, 26]. After PCI, functionally active neutrophil infiltration is aggravated locally in the balloon-injured arteries [27]. Endothelial activation and expression of adhesion molecules such as selectins and intercellular adhesion molecule (ICAM)-1 aggravate adhesion and recruitment of neutrophils [28]. Locally infiltrated neutrophil mediates destabilization of atherosclerotic plaques [29]. Owing to the anatomic proximity to the coronary arteries, metabolically active EAT has an additional atherogenic inflammatory effect on culprit lesions [2, 7]. The paracrine and vasocrine secretions of inflammatory adipokines by EAT, such as IL-1, IL-6, ICAM, tumor necrosis factor-α and nerve growth factor, contributes to the amplification of vascular inflammation [7]. Although plasma inflammatory biomarkers might not adequately reflect local inflammation, the presence of inflammatory adipokines by EAT might reflect the response to plaque rupture and perivascular inflammation adjacent to atherosclerotic lesions [30]. Through additional local vascular inflammation, the presence of metabolically active adipose stores that surround epicardial coronary arteries could contribute toward the adverse clinical outcomes in patients with STEMI.

The present study has several limitations. First, echocardiography is not the optimal methods for exact quantification of total EAT. Although EAT thickness by echocardiography does not exactly represent the amount of total EAT, EAT thickness by echocardiography correlates well with total EAT measured by computed tomography or magnetic resonance imaging [11, 31]. The majority of clinical studies have reported excellent intra- and inter-observer agreement for the measurement of EAT thickness by echocardiography on the parasternal long-axis view [1, 11–13, 31]. Although there is still debate of echocardiographic measurement techniques and some studies have measured EAT thickness at end-systole [14], EAT thickness by echocardiography at end-diastole was well correlated with total amount of EAT in our previous studies [1, 11]. Measurement of EAT thickness by echocardiography might be reliable and relatively accurate. Second, a normal-cut off value of EAT thickness by echocardiography has not yet been established. As ethnic differences could influence the distribution of EAT [32], the cut-off value of EAT thickness by echocardiography for predicting adverse clinical outcomes might be different according to the ethnicity. Also, there is possibility of gender difference, as estrogen status could affect the accumulation of visceral adipose tissue [33]. In the present study, we could not demonstrate the gender difference in the relationship between EAT and clinical outcomes, owing to relatively small sample size of females with MACEs. Further studies might be needed for clinical application.

## Conclusions

This study demonstrates that the EAT thickness is related with long term clinical outcome in patients with STEMI. The EAT thickness might provide additional information for future clinical outcome, especially TVR.

## Abbreviations

CAD: Coronary artery disease
CI: Confidence intervals
EAT: Epicardial adipose tissue
HR: Hazard ratio
MACEs: Major cardiac adverse events
PCI: Percutaneous coronary intervention
STEMI: ST elevation myocardial infarction
TIMI: Thrombolysis in myocardial infarction trial
TVR: Target vessel revascularization

## References

[1] Ahn SG, Lim HS, Joe DY (2008). Relationship of epicardial adipose tissue by echocardiography to coronary artery disease. Heart.

[2] Park JS, Choi SY, Zheng M (2013). Epicardial adipose tissue thickness is a predictor for plaque vulnerability in patients with significant coronary artery disease. Atherosclerosis.

[3] Park JS, Choi BJ, Choi SY (2013). Echocardiographically measured epicardial fat predicts restenosis after coronary stenting. Scand Cardiovasc J.

[4] Husser O, Bodi V, Sanchis J (2011). White blood cell subtypes after STEMI: temporal evolution, association with cardiovascular magnetic resonance--derived infarct size and impact on outcome. Inflammation.

[5] Anzai T, Yoshikawa T, Takahashi T (2003). Early use of beta-blockers is associated with attenuation of serum C-reactive protein elevation and favorable short-term prognosis after acute myocardial infarction. Cardiology.

[6] Carrick D, Haig C, Rauhalammi S (2015). Pathophysiology of LV remodeling in survivors of STEMI: inflammation, remote myocardium, and prognosis. JACC Cardiovasc Imaging.

[7] Iacobellis G, Bianco AC (2011). Epicardial adipose tissue: emerging physiological, pathophysiological and clinical features. Trends Endocrinol Metab.

[8] Iozzo P (2011). Myocardial, perivascular, and epicardial fat. Diabetes Care.

[9] Lo J, Abbara S, Rocha-Filho JA, Shturman L, Wei J, Grinspoon SK (2010). Increased epicardial adipose tissue volume in HIV-infected men and relationships to body composition and metabolic parameters. AIDS.

[10] Bulkley BH, Roberts WC (1975). The heart in systemic lupus erythematosus and the changes induced in it by corticosteroid therapy. A study of 36 necropsy patients. Am J Med.

[11] Hwang JW, Choi UJ, Ahn SG (2008). Echocardiographic plains reflecting total amount of epicardial adipose tissue as risk factor of coronary artery disease. J Cardiovasc Ultrasound.

[12] Iacobellis G, Assael F, Ribaudo MC (2003). Epicardial fat from echocardiography: a new method for visceral adipose tissue prediction. Obes Res.

[13] Iacobellis G, Willens HJ, Barbaro G, Sharma AM (2008). Threshold values of high-risk echocardiographic epicardial fat thickness. Obesity (Silver Spring).

[14] Iacobellis G, Willens HJ (2009). Echocardiographic epicardial fat: a review of research and clinical applications. J Am Soc Echocardiogr.

[15] Park JS, Ahn SG, Hwang JW (2010). Impact of body mass index on the relationship of epicardial adipose tissue to metabolic syndrome and coronary artery disease in an Asian population. Cardiovasc Diabetol.

[16] Thygesen K, Alpert JS, White HD (2007). Universal definition of myocardial infarction. Circulation.

[17] Puymirat E, Simon T, Steg PG (2012). Association of changes in clinical characteristics and management with improvement in survival among patients with ST-elevation myocardial infarction. JAMA.

[18] Michaels AD, Goldschlager N (2000). Risk stratification after acute myocardial infarction in the reperfusion era. Prog Cardiovasc Dis.

[19] Eagle KA, Lim MJ, Dabbous OH (2004). A validated prediction model for all forms of acute coronary syndrome: estimating the risk of 6-month postdischarge death in an international registry. JAMA.

[20] Christia P, Frangogiannis NG (2013). Targeting inflammatory pathways in myocardial infarction. Eur J Clin Invest.

[21] Arslan F, Smeets MB, O’Neill LA (2010). Myocardial ischemia/reperfusion injury is mediated by leukocytic toll-like receptor-2 and reduced by systemic administration of a novel anti-toll-like receptor-2 antibody. Circulation.

[22] Bianchi ME (2007). DAMPs, PAMPs and alarmins: all we need to know about danger. J Leukoc Biol.

[23] Timmers L, Sluijter JP, van Keulen JK (2008). Toll-like receptor 4 mediates maladaptive left ventricular remodeling and impairs cardiac function after myocardial infarction. Circ Res.

[24] Bujak M, Dobaczewski M, Chatila K (2008). Interleukin-1 receptor type I signaling critically regulates infarct healing and cardiac remodeling. Am J Pathol.

[25] De Servi S, Mazzone A, Ricevuti G (1995). Clinical and angiographic correlates of leukocyte activation in unstable angina. J Am Coll Cardiol.

[26] Jin SA, Seo HJ, Kim SK (2015). Elevation of the serum apurinic/apyrimidinic endonuclease 1/redox factor-1 in coronary artery disease. Korean Circ J.

[27] Welt FG, Edelman ER, Simon DI, Rogers C (2000). Neutrophil, not macrophage, infiltration precedes neointimal thickening in balloon-injured arteries. Arterioscler Thromb Vasc Biol.

[28] Palazzo AJ, Jones SP, Anderson DC, Granger DN, Lefer DJ (1998). Coronary endothelial P-selectin in pathogenesis of myocardial ischemia-reperfusion injury. Am J Physiol.

[29] Naruko T, Ueda M, Haze K (2002). Neutrophil infiltration of culprit lesions in acute coronary syndromes. Circulation.

[30] Mazurek T, Zhang L, Zalewski A (2003). Human epicardial adipose tissue is a source of inflammatory mediators. Circulation.

[31] Iacobellis G, Ribaudo MC, Assael F (2003). Echocardiographic epicardial adipose tissue is related to anthropometric and clinical parameters of metabolic syndrome: a new indicator of cardiovascular risk. J Clin Endocrinol Metab.

[32] Willens HJ, Gómez-Marín O, Chirinos JA, Goldberg R, Lowery MH, Iacobellis G (2008). Comparison of epicardial and pericardial fat thickness assessed by echocardiography in African American and non-Hispanic White men: a pilot study. Ethn Dis.

[33] Ibrahim MM (2010). Subcutaneous and visceral adipose tissue: structural and functional differences. Obes Rev.

